# Rhyme and reason: the role of circadian rhythms in skin and its implications for physicians

**DOI:** 10.4155/fsoa-2016-0007

**Published:** 2016-04-06

**Authors:** Kavita Beri, Sandy Saul Milgraum

**Affiliations:** 1 Center For Dermal Research, Department of Biomaterials, Rutgers – The State University of New Jersey, Life Sciences Building, 145 Bevier Road, Piscataway, NJ, 08854 USA; 2 Division Of Dermatology, Robert Wood Johnson Medical School, New Brunswick, NJ, 08901 USA

**Keywords:** cell cycle, circadian rhythm, epidermal stem cells, skin

The study of the day and night cycle and its effects on the human body has been a field of interest for many decades. In more recent years, the effects of these diurnal variations have been looked into with respect to human skin. To encourage future medical interest in this subject as well as to discuss the futuristic concept of transdermal drug delivery incorporating the effects of circadian rhythms, we have summarized recent research into this topic.

The day and night cycle creates widely different environments for the skin. Depending on the time of day, there is variation in the risk of UV exposure, transdermal water loss and exposure to extremes of temperature. Diurnal rhythms are patterns that recur every 24 h, whereas circadian rhythms or biorhythms persist with a period of ˜24 h in the absence of external cues but remain responsive to environmental conditions such as light and exercise [1]. The circadian clock, a primordial system, which regulates human physiology based on diurnal variation, has a natural impact on the way our skin functions. In plants, the circadian control of development is well established. Research in recent years has focused on variations of the circadian rhythm in human skin and its impact on its integral complex components [2]. In the following report we summarize recent research on circadian oscillations and its effect on the skin. Human circadian cues originate in the suprachiasmatic nucleus (SCN). The SCN regulates the timing of body clocks via autonomous nervous control of hormones such as glucocorticoids or by sending indirect signals via body temperature. The SCN is itself synchronized to light via the retinohypothalamic tract; the result is a flexible system of clocks, each with an intrinsic period of about one day that is constantly readjusting to the timing of environmental light [3].

## Functioning of the clock in the skin

This molecular basis of the circadian clock involves feedback loops of transcription and translation that regulate cAMP levels within the cell and thus various protein kinases [3]. The clock proteins CLOCK and BMAL1 activate the transcription of other clock genes, for example, Per1–Per3 and the cryptochromes Cry1 and Cry2, the protein products of which repress their own transcription by blocking CLOCK:BMAL1 mediated activation [4]. New evidence suggests that clock regulations in the skin functions may have a more robust intrinsic clock influence and not just the control of SCN [5]. These function more like multiple independent, but coordinated, peripheral clocks that work within distinct anatomic compartments of the skin [6]. There is a robust circadian clock in the stem cells of the basal epidermal cells and keratinocytes. Experiments involving the deletion of clock genes in this germline show that the circadian rhythms modulate diurnal cycles of DNA repair and replication [2]. In addition to cell cycle regulation circadian rhythms have also been related to stem cell senescence and tissue repair processes [6,7]. Hair graying, loss of subcutaneous fat layer and delayed tissue healing are some consequences of skin aging [2]. *BMAL1* deficient mouse models have shown premature aging and thereby suggest an influence of circadian clocks on cellular aging [7].

## Circadian oscillations & epidermal stem cells

Recent research shows that circadian clocks can play an important role in human regenerative processes that can be faster or slower than the biological day [3]. UVB penetration of the skin is largely in the interfollicular epidermis where circadian cell gating is prominent [3]. In humans the maximum number of epidermal progenitors go through S phase of the cellular cycle during the day, which is the time for maximum sun exposure [3]. During the hours of exposure to light, two different consecutive cohorts of genes are predominantly involved in the protection from UV, and in inducing proliferation to reach its peak [2,7–8]. Thus human keratinocytes are able to anticipate their need for protection against UV at the time they are most active in synthesis of DNA [9]. A few papers have shown that both mature cells and pluripotent stem cells respond to oscillation of circadian rhythms, but its exact role and effect on their differentiation needs to be further investigated [1]. Janich *et al*. [9] also showed that the oscillations of the core clock transcriptional machinery consists of successive waves along the 24 h of the day. Shifts of every 4–5 h were observed that defined a continuum of functional landmarks in the vital functions in the keratinocytes. They predisposed the undifferentiated keratinocytes to respond to differentiation cues between the late night and morning transition. In proliferating stem cells, the circadian clock coordinates activities of oxidative phosphorylation and glycolysis with DNA synthesis, perhaps as a protective mechanism against genotoxicity [10,11].

## Circadian oscillations & cancer stem cells & chemotherapy

Use of electric lights in the modern era predisposes people to prolonged light exposure throughout the year. This disruption in circadian oscillation may induce a wide variety of stresses on cells [1,4]. Circadian genes have been shown to function as oncogenes or tumor suppressors at systemic or cellular levels through their involvement in cell proliferation, cell cycle regulation, apoptosis and DNA damage signaling, indicating a direct circadian effect on cancer cells [12]. Clinical tumors comprise a heterogeneous population of cells that include cancer stem cells [12]. These cancerous stem cells are greatly dependant on their niches (microenvironments) for growth and stability [13]. WNT10a is the key molecule in the development of tumor niches [14]. Circadian rhythm influences chemotherapy in cancer patients by affecting cell cycle, DNA repair and tissue metabolic enzymes that are involved in drug sensitivity [15]. The tumor microenvironment is crucial for solid tumor development. Dysregulation of Ph and disruption of tumor niche is closely involved in the cancer hallmark of invasion and metastasis [16]. These observations also suggest that circadian disruption in cancer patients might be related to poor prognosis [8]. Chronotherapy is a new approach that explores the administration of therapy at specific times of the day to maximize benefit and reduce side effects [8,9]. Circadian variation of drug metabolizing enzymes in the skin and the effect on transdermal water loss has been researched, and suggests that diurnal variation in epidermal barrier functions can be explored further to improve transdermal drug delivery [8].

## Circadian rhythms & the future of regenerative medicine

The role of the circadian clock within the skin needs further exploration, simplistically considering a singular clock mechanism, or ‘skin clock’ can help explain the physiological role circadian variations have on the continuously dividing skin cells [6]. Elucidation of circadian oscillation could lead to optimizing therapy in wound healing and better grasping the aging cell. Future research in regenerative medicine can focus on studying oscillations within the epidermal germline and might hold the key to unlocking secrets that influence the regeneration of the skin.
